# Thermal and environmental analysis of Cucumis sativus drying in a mixed mode solar dryer with combined sensible and latent heat energy storage

**DOI:** 10.1038/s41598-025-91971-4

**Published:** 2025-03-05

**Authors:** C. N. Deepak, Aruna Kumar Behura

**Affiliations:** School of Mechanical Engineering, Vellore Institute of Technology, Vellore, 632014 TN India

**Keywords:** Mixed mode solar dryer, Energy, Exergy, Thermal energy storage, Sustainability, CO_2_ mitigation, Energy harvesting, Energy storage, Renewable energy

## Abstract

This study presents a thermodynamic analysis of a mixed-mode solar dryer incorporating both sensible and latent heat energy storage materials. Black pebbles were utilized for sensible heat storage, while Lauric acid was selected for latent heat storage. The integration of these energy storage materials significantly enhanced the thermodynamic performance of the dryer, achieving a peak energy efficiency of 14.2% and a 53% increase in average energy efficiency. Additionally, the inclusion of latent heat storage in the collector resulted in the highest recorded collector energy efficiency of 84.6%. Exergy analysis indicated a maximum exergy efficiency of 51.3%, with an average exergy efficiency of 34.3% for the dryer. The implementation of combined thermal energy storage led to a 50% reduction in drying time. Sustainability assessments demonstrated that integrating both sensible and latent heat storage improved energy utilization while minimizing losses, thereby enhancing the overall sustainability and productivity of the solar dryer.The environmental analysis estimated a CO₂ mitigation potential of 83.97 tonnes per year, with a corresponding carbon credit value of $419.85. The system exhibited a remarkably low energy payback period of 1.82 years when operated with both thermal energy storage materials. This research underscores the potential benefits of combining latent and sensible heat storage in solar drying applications, highlighting its contribution to sustainability and the environmental advantages of solar thermal systems.

**Supplementary Information:**

The online version contains supplementary material available at 10.1038/s41598-025-91971-4.

## Introduction

Dehydration has been an effective and primitive technique for enhanced shelf life of perishable agricultural products.The moisture content present in agro products favour microbial growth leading to their degradation making it difficult for their harvesting, handling, transport and storage^1–3^.When these products are subjected to drying, they are preserved for a much longer period while retaining their nutritional quality and will lead to reduced post-harvest losses^4^. Drying is an energy intensive operationwhich spends 12–15% of the entire industrial energy consumption, and is an integral activity of many industrial processes^5–7^.When this energy demand is satisfied through conventional energy resources such as fossil fuels, the entire process turns out to be detrimental for the environment along with high cost of operation^8–12^.For a sustainable drying operation, the entire process needs to be carried out through renewable energy resources, which reveals the potential and significance of solar dryer technology^11–14^. Being the most abundantly available primary source of renewable energy, utilizing solar energy for drying operations can have definite environmental and economic advantages^8,15,16^.

The conventional approach to utilizing solar energy for drying is open-sun drying, where products are spread over a large surface area and directly exposed to solar radiation. While this method is environmentally friendly, it has several drawbacks. Products left in the open are vulnerable to contamination from dust, microbes, rodents, and insects, compromising hygiene and safety. Additionally, exposure to fluctuating weather conditions can lead to extended drying times and reduced product quality. The unpredictability of sudden rainfall, the labour-intensive nature of the process, and the inability to operate during non-sunshine hours further limit the efficiency and reliability of open-sun drying^17,18^.These disadvantages have been successfully tackled through the development of solar dryers, where the products are placed in confined chambers without being exposed to outside environment, but enables reception of the thermal energy from incoming solar irradiance. Solar dryers are environment friendly, economically viable solar thermal devices which harness the thermal energy from the solar irradiancethat falls on it through various means depending on the nature and design of the same^19^. Their major classification is based on their interaction with solar energy, in which direct solar dryers are transparent structures which expose the products placed inside it towards incoming solar irradiance and traps the incoming heat inside the dryer for elevated drying temperature. Indirect mode solar dryers use solar energy as an auxiliary source of heating where an external collector connected to the dryer suppliessolar heated air into the dryer and theproducts are not exposed to sunlight.In Mixed mode drying, both direct and indirect dryer designs are combined where solar thermal energy is used both directly and as an auxiliary source with an external collector^20^.

Over the years, the design and performance of the solar dryers wereexplored by researchers for different nature and types of products and have also reported remarkable results towards drying performance and overall effectiveness of the technology.Nabnean et al. analysed the thermal characteristics of a direct mode solar dryer loaded with banana slices. The dryer could hold 10 kg of bananas which was dried in under 4 days a drying time that was 48% lessagainst natural open-sun drying. A highest drying temperature of 60 °C was observedinside the dryer during the experiment^21^. Hidalgo et al. designed and fabricated a direct mode solar dryerloaded with green onion. An average drying efficiency of 38.3% was observed in the study. A better retention of colour was also reportedfor products dried in the solar dryer^22^. Ndukwu et al. compared the drying characteristics of a direct solar dryer under three different collector cover colours. Neem leaves were chosen as the product for the experiment and yellow colour collector cover exhibited the best results with 38.8% thermal efficiency, higher temperature and lower drying time^23^. Islam et al. investigated how the design of the cover influences the effectiveness of a cabinet-type direct solar dryer.Four different fruits were dried for the experimental analysis. Natural draft type chamber exhibited the best results with a moisture removal rate of 58.9%^24^.Salhi et al. studied the drying characteristics of an indirect solar dryerloaded with tomatoes. The dryer temperature went up to 73 °C and uniform drying of tomatoes were observedwithin 7 h^25^. Salhi et al. explored how the type of drying trays affects the performance of an indirect solar dryer loaded with banana slices. The highest drying air temperature was 60 °C and the highest drying rate was observed in a wire mesh drying tray^26^. Ennisioui et al. investigated the thermal characteristics of an indirect solar dryer loaded with banana slices. The dryer temperature reached a maximum of 58 °C, with the highest reported dryer efficiency at 18.8%.After 9 h of drying time, the solar dryer resulted in a significantly lower moisture content in the products compared to open sun drying^27^. Verma et al. studied the performance of a mixed mode solar tent dryer with potato slices loaded in it. A highest dryer temperature of 59.7 °C was recorded and a reduction in drying time by 6 h. The dryer reported a maximum efficiency of 26.62%^28^.Ekka et al. dried cluster figs in a mixed mode solar dryer integrated with two double pass solar air heaters.41.4% of exergy efficiency was achieved by the drying chamber when operated with an air flow rate of 3.72 kg/min^29^. Doris et al. studied the drying kinetics and environmental characteristics of a mixed mode solar dryer with blanched potato slices as the product. A 12-hour reduction in drying time was observed with a collector efficiency of 34%. The dryer had a CO_2_ mitigation potential of 12.42 tonnes per year and an energy payback period of 0.44 years^30^.Murugavelh et al. dried tomato waste in a mixed mode solar tunnel dryerhaving a solar air heater connected to it. With the highest dryer temperature of 67 °C,7 h drying time was reported by the dryer, compared to 15 h under open sun^31^.

Even though solar dryers are versatile, ecofriendly and achieve enhanced drying rate and quality retention of dried products, one of their major limiting factors is the reliance to solar irradiance availability and favourable climatic conditions, which restricts their reliability and operation hours. The non-sunshine hours and variations in the weather such as cloudy sky limit the working of solar dryers. This can be effectively tackled using an efficient thermal energy storage system, where excess energy available during the day is stored and is utilized during low to non-sunshine hours^32^. Thermal energy storage materials are majorly classified into sensible heat (SHES) and latent heat (LHES) energy storage materials. SHES store energy within them by increasing their temperature, which is later retrieved. Materials such as sand, gravels, pebbles, rock bed, thermal oils, water etc. are usually employed as SHES materials^33^. LHES materials utilize the incoming energy for an isothermal phase transition, which enables them to store excessive amount of energy within low volume of material^34^.Sundari et al. used a bed of gravel laid on the dryer base for SHESand papaya slices were loaded in the dryer for experimental evaluation. The provision for energy storage resulted in reduced drying time and an effective moisture diffusivity of 6.15303 × 10^− 8^ m^2^/s was achieved in the dryer^35^. Vijayan et al. used pebbles for SHES in an indirect solar dryer where bitter gourd slices were used as the product. Effective drying was achieved within 7 h and the dryer system had an efficiency of 19%. The highest moisture extraction rate achieved was 0.215 kg/kWh under an air flow rate of 0.0636 kg/s^36^.Qui˜nones et al. studied and compared the thermal characteristics of an indirect solar dryer with tomato slices, having limestone and beach sand as SHES materials. The dryer system with limestone exhibited better moisture removal rate and efficiency in the study. Even though beach sand stored more energy within it, its susceptibility to solar radiation and weather conditions led to a decline in the dryer performance^37^. Andharia et al. used black pebbles for energy storage in a mixed mode solar dryer loaded with shrimps. SHES integration resulted in elevated temperature output from the collector 7 h post sunshine. The dryer had a thermal efficiency of 25.47% and the dried shrimp from the dryer with SHES had better quality and acceptability^38^. Murugesan et al. assessed the drying characteristics of a solar dryer havingPCM placed on inner walls of drying chamber. Paraffin wax in copper tubes were placed in vertical and horizontal orientation in the drying chamber which was loaded with banana slices. The experimental study compared the drying characteristics with PCM loaded at two distinct quantitates and the larger quantity of PCM led to a decrease in drying time by 6 h.The dryer system had a maximum exergetic efficiency of 28%^39^. Chaatouf et al. conducted a similar study on an indirect solar dryer having paraffin wax filled copper tubes placed inside a plenum chamber mounted on the side. Orange slices used as the product and the provision of LHES reduced the dryer time by 24 h. A higher vitamin C content was also observed in the orange slices dried in the solar dryer with PCM^32^.Madhankumar et al. examined the drying characteristics of bitter gourd slices using an indirect solar dryer with LHES. High thermal conductivity aluminium fins were inserted into PCM which resulted in better results in terms of energy capture.The LHES resulted in a 4 h reduction in drying time and a19.6% dryer efficiency was observed^40^.

The literature review clearly indicates that incorporating thermal energy storage materials has led to substantial performance enhancements in solar dryers, in terms of extended working hours and lowered drying time. The SHES capacity of different naturally available materials has been explored such as stone chips, rocks etc. while paraffin wax is the most common LHES material. From the limited literature review of the authors, the energy and exergy characteristics of solar dryers integrated with either SHES or LHES have been analysed and studied extensively^41–47^while the possibilities and effects of integratingboth SHES and LHES materials in a solar dryer system is yet to be explored, leaving extensive research gaps. Considering the evident advantages of energy storage provision in previously reported studies, a possibility towards availing the compound benefit of both thermal energy storage materials could be highly advantageous for a solar dryer. Combined thermal energy storage in a solar dryer require strategic placement of the materials along with consideration towards quantity and ease of integration.Even though LHES by paraffin wax has reported desirable performance enhancement results, there are still numerous LHES materials with high energy density and desirable melting temperature that are to be investigated for their practical implementation in solar dryers. It is also observed that there are very few studies which undertook the sustainability aspect of solar dryers and analyse them using the exergy sustainability indicators. The placement of energy storage materials is as crucial as the nature and quantity of them and from the literature review, most of the experimental set up have their energy storage especially SHES materials placed on the solar collector rather than the drying chamber, which is prone to increased losses during transmission. Solar dryers withLHES have the energy storage materials either placed within the drying chamber or cascaded inside the solar collector structure. Mixed mode solar dryers have the luxury of using incoming solar irradiance as direct as well as auxiliary source, which provides even more opportunities for energy capture within their structure and placement of energy storage.

The study aims to integrate both SHES and LHES on a mixed mode solar dryer system, analysing the energy and exergy characteristics whileCucumis sativus slices were used as the product. The SHES material is placed within the drying chamberwhile the LHES material is integrated into the dryer system through a novel PCM chamber connected at the collector outlet.The dryer assembly consisted of a solar tunnel dryer having transparent walls equipped with flat plate solar collector having a blower at its inlet.Under a constant flow rate, the experimental analysis was carried out in real time with equal quantities of cucumber slices dried in the solar dryer under three different cases. Initially the solar dryer was operated without any thermal energy storage materials which is denoted as case 1.Further, the SHESmaterial was laid down at the base of the drying chamber and the experiment was repeated which is case 2.In the final case of experiment, both SHES and LHES materials were integrated into the dryer and the drying operation was carried out, which is denoted as case 3.The study investigates the potential of a mixed-mode solar dryer system in harnessing incident thermal energy and the impact of adding combined thermal energy storage, through thermodynamic, sustainability, and environmental analyses. A sustainability perspective is presented through suitable indices andenvironmental impact of the dryer through integration of combined energy storage was highlighted in the studyby examining the embodied energy, energy pay-back period and CO_2_ mitigation potential.

## Materials and methods

### Experimental procedure

**Fig. 1:**
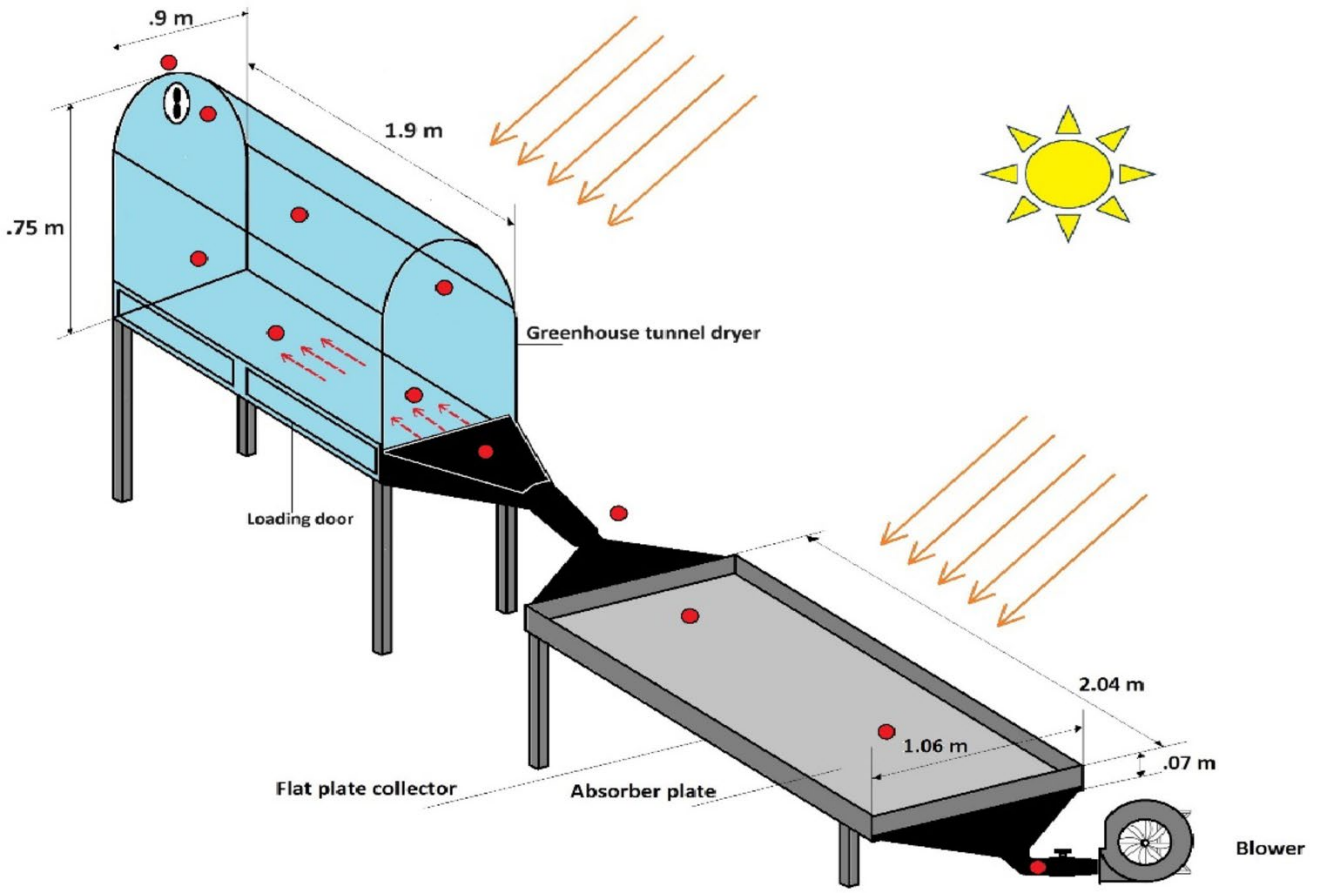
Mixed mode solar dryer experimental set up schematic diagram.

The study explores the possibilities and effects of integrating both SHES and LHES in a mixed mode solar dryer loaded with Cucumis sativus. The dryer was operated on the roof top over an area with no chances of shading and the dryer system was cleaned before and after every set of experiment.Lauric acid PCM was integrated into the dryer assembly for LHES through a novel trapezoidal heat exchanger, while polished black pebbles were used as the SHES material which was laid on the dryer bed. The experiment was carried out from 9 AM to 6 PM and the temperature profile of the solar collector and solar dryer was constantly monitored and recorded using thermocouples placed at different locations throughout the dryer. The mass flow rate was maintained at constant value of 0.048 kg/s and the solar irradiance during the experimental hours were measured and recorded. The samples were sliced in at 2 mm thickness and spread evenlyover two wire mesh drying trays and were loaded inside the drying chamber through loading doors on the side. The weight of the samples was measured hourly and for experiments that extend to the next day, the samples were placed inside an air tight container overnight.The schematic diagram of dryer assembly is presented in Fig. 1.

### Mixed mode solar tunnel dryer

#### Solar collector

**Fig. 2:**
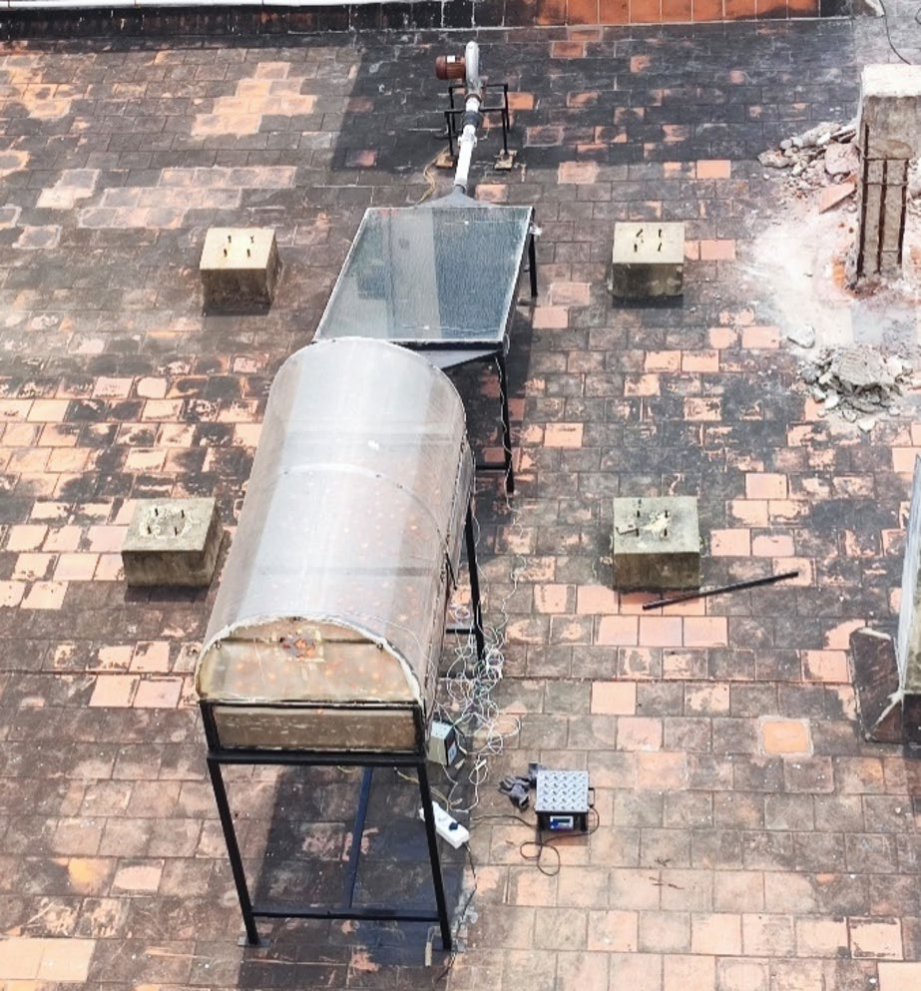
Solar tunnel dryer.

An underflow solar air heater whichwas 2.04 m long and 1.06 m wide was used for heated air input into the drying chamber, as seen in Fig. 2. It had a single transparent glass glazing and a 2 m long and 1.04 m wide aluminium sheet absorber plate with diverging and converging triangular sections at the inlet and outlet respectively for even distribution ofheated air.The centrifugal blower placed at the inlet ensured a forced convection under constant mass flow rate. The collector was placed on an iron frame with a 30° inclination and the outlet was connected to the bottom of drying chamber using insulated PVC pipes. The bottom of solar collector was insulated with EPE foam sheet.

#### Solar tunnel dryer

**Fig. 3:**
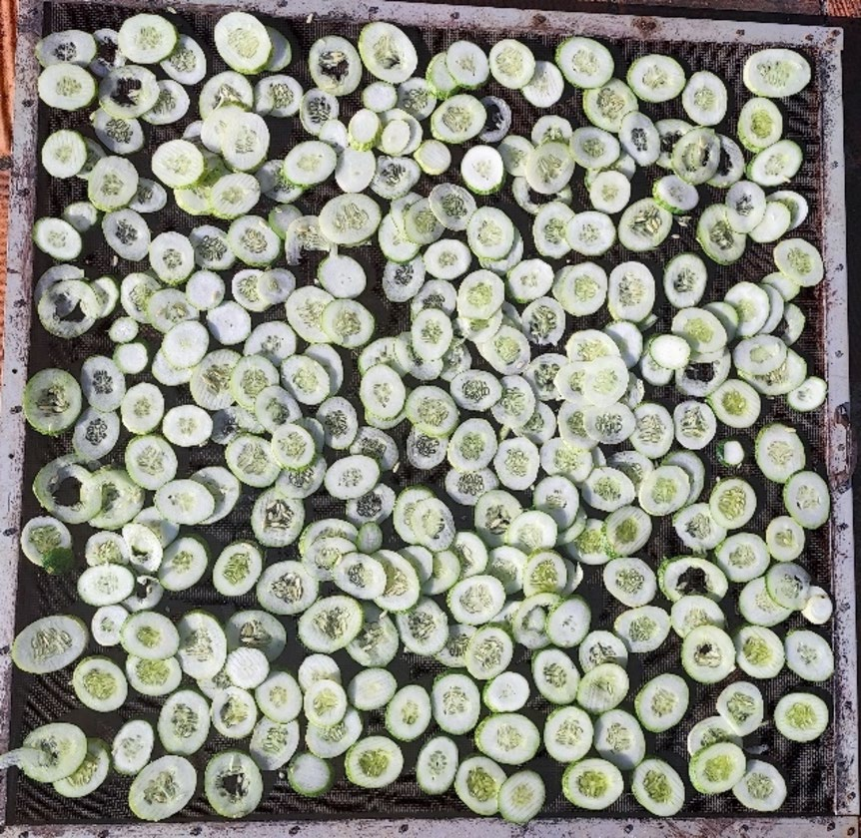
Fresh Cucumis sativus sliced and loaded into the drying tray.S

Drying chamber of the solar dryer system was 1.9 m long and 0.9 m wide which was fabricated using UV stabilized transparent polycarbonate sheets mounted on a 0.45 m radius hemispherical shaped mild steel frame, as shown in Fig. 3. Two drying trays, 0.83 m long and 0.78 m wide, made of aluminium frame and thick wire mesh were fabricated to hold the product without sagging. An exhaust fan was provided on top of north wall to remove drying air with high moisture content. The transparent walls and curved design on top ensured maximum reception of incoming solar irradiance by the products. A trapezoidal extended surface at the solar collector outlet diverged into the drying chamber base, facilitating even distribution of the heated air over the products.

#### Cucumis sativus

It is one of the most popular vegetables in the world with high minerals, anti-oxidants and vitamins. The high moisture content and polysaccharides present within them leads to their deformity and spoilage during post-harvest, and are also prone to damages and rotting while handling them^48^. Dehydration serves to be a proper preservation technique for Cucumis sativus in prolonged shelf life, while retaining their qualities. In this experimental study, 4 kg of Cucumis sativus was used as the product which had an initial moisture content of 94%. They were purchased fresh from the local market whichwere washed and sliced at 2 mm thickness and spread evenly over the wire mesh drying traysasseen Fig. 2.

#### Thermal energy storage

**Fig. 4:**
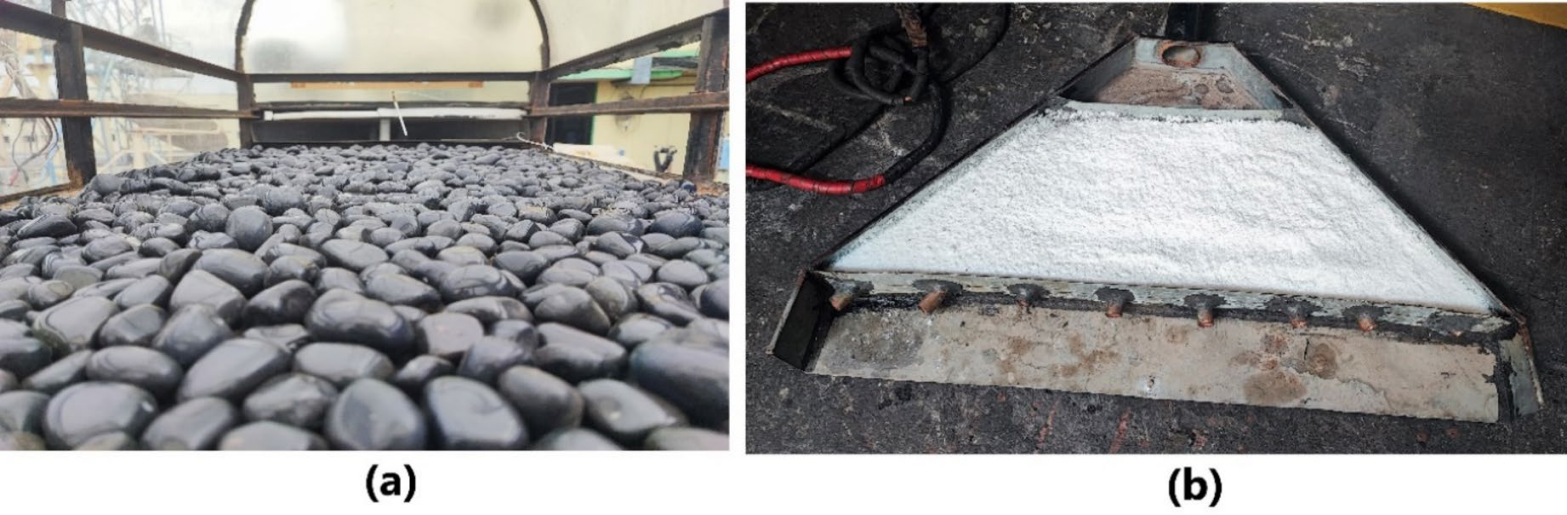
(**a**) Black pebbles on the dryer bed for sensible heat energy storage and (**b**) Lauric acid filled inside the PCM chamber for latent heat energy storage.

For sensible heat energy storage, black pebbles with polished surface were chosen in the experiment. 20 kg of black pebbles were evenly spread across the base of drying chamber, allowing them being exposed to the solar collector output and incoming direct solar irradiance, as seen in Fig. 4a. An organic fatty acid PCM of Lauric acid was filled in a custom-built trapezoidal heat exchanger as LHES material. The suitability in melting point and high latent heat of fusion made it a desirable energy storage material, which was yet to be explored in solar thermal applications. Table 1 presents the thermophysical characteristics of Lauric acid. 4 kg of Lauric acidhaving a melting point of 40.86 °C was filled inside PCM chamber as seen in Fig. 4b, which was connected at the solar collector outlet diverging towards drying chamber base.The PCM chamber was built using sheet metal which was selectively coated on the outside and was made leak proof inside by using industrial sealant and silicon glue. 8copper tubes of 0.01 m diameter ran through the length of the chamber for hot air to pass through while lauric acid was filled over the tubes, immersing them inside it. This mimicked heat transfer mechanism of a shell and tube heat exchanger between hot air and PCM along with heat collection from direct solar radiation through the exposed outer area of the chamber. The dimensions and specifications of different components in the dryer system including the solar collector, drying chamber and thermal energy storage unit is listed out in Table 2.

**Table 1:** Thermophysical properties of lauric acid PCM.

Organic PCM	IUPAC Name	Melting point (°C)	Latent heat of fusion (J/g)	Freezing point (°C)	Latent heat of freezing (J/g)	Density (kg/m^3^)
Lauric Acid	Dodecanoic acid	40.86	196.95	36.76	205.75	880

**Table 2:** Dimensions of different components of solar dryer.

S. no.	Component of dryer	Dimensions and materials
Drying chamber		
1	Length	1.9 m
2	Width	0.9 m
3	Radius of hemisphere	0.45 m
4	Volume inside dryer	1.12m3
5	Covering material	UV stabilized Polycarbonate sheet
6	Cover thickness	1.5 mm
7	Drying tray size	0.80*0.83 m
8	Base of dryer	Stainless steel
9	Insulation material	EPE foam sheet
10	Insulation thickness	5 cm
Solar collector		
1	Length	2.04 m
2	Width	1.06 m
3	Height	0.07 m
4	Absorber plate	Aluminium sheet
5	Air passage width	3 cm
6	Insulation material	EPE foam sheet
7	Insulation thickness	5 cm
8	Glazing material	Transparent glass
9	Glazing thickness	8 mm
10	Blower	250 W, 220 V, 50 Hz, 1380 rpm
11	Tilt angle	30°
PCM chamber		
1	PCM chamber material	Stainless steel sheet metal
2	PCM chamber base length	0.33 m
3	PCM chamber side length	0.21 m
4	PCM chamber height	0.245 m
5	Copper tube diameter	1 cm
6	Number of copper tubes	8
7	Sealing material	Silicone adhesive
8	Insulation material	EPE foam sheet
9	Insulation thickness	5 cm

### Instrumentation

The solar irradiance of the location was measured and recorded using a Hukseflux SR11 pyranometer with a range of up to 3000 W/m^2^. Pt 100 thermocouple sensors measured the temperature over various locations ofdrying chamber and solar collector. Temperature sensor probes were fastened at every location and then connected to a16-channel data logger. The measured data were recorded at equal intervals over the entire span of the experiment by the data logger. A Lutron-AM 4204 hot wire anemometer measured the air flow rate from the blower. The products were weighed using a TVS portable weighing machine of 50 kg capacity. The detailed specification and accuracy of instruments used for the research is given in Table 3.

**Table 3:** Technical specifications, range and accuracy of the instruments.

Sl no.	Instrument	Specification	Accuracy	Device images
1.	Data logger	16 channel digital scanner, Model 6000, Range 0-200ºC, 230 V AC	± 0.3%	
2.	Temperature sensor	Pt 100 RTD sensor, 6 × 5 inch, -200 to 850 ºC	± 0.015ºC	
3.	Weighing machine	TVS brand portable scale, 50 kg capacity	± 0.5 g	
4.	Anemometer	Lutron-AM 8102, Vane type anemometer, 0.2 to 20 m/s	± 5%	
5.	Pyranometer	Hukseflux SR11, class A ,0 to 3000 W/m², 285 to 3000 nm, < 1% temperature sensitivity	± 1%	

### Energy analysis

The energy analysis is conducted considering a uniform mass flow rate throughout and the minor variations are neglected. The solar collector receive energy directly from the incoming solar irradiance and the drying chamber receives direct solar energy as well as hot air output from solar collector. Energy losses occur over the walls and joints of drying chamber, PCM container, unabsorbed heat by absorber plate etc. The analysis is performed considering conservation of mass and energyprinciple^49^.

1 $$\:\sum\:{\dot{m}}_{in}=\sum\:{\dot{m}}_{out}$$

Considering the conservation of energy equation,

2 $$\:Q-W=\sum\:{\dot{m}}_{out}\left({h}_{o}+\frac{{{v}_{0}}^{2}}{2}+{z}_{o}g\right)-\sum\:{\dot{m}}_{in}\left({h}_{in}+\frac{{{v}_{i}}^{2}}{2}+{z}_{i}g\right)$$

where the net heat transfer into dryer system is denoted by$\:Q$, $\:W$ denotes net work done by system. $\:h$ denotes enthalpy, $\:v$ denotes the velocity and $\:z$ represents the height from the reference.

#### Energy analysis of the solar collector

$\:{Ec}_{in}$represents thetotal energy incident on solar collector, which is given by

3 $$\:{Ec}_{in}=\tau\:\times\:\alpha\:\times\:I\times\:{A}_{c}$$

Where $\:\tau\:$ and $\:\alpha\:$ are the transmittance and absorptance values which are 0.85 and 0.95 respectively.

$\:{Q}_{c}$ represents the useful energy gain by the solar collector, given as

4 $$\:{Q}_{c}=\dot{{m}_{a}}\times\:{C}_{p}\times\:({T}_{co}-{T}_{ci})$$

The energy efficiency of the solar collector is given by

5 $$\:{\eta\:}_{c}=\frac{{Q}_{c}}{{Ec}_{in}}$$

#### Energy analysis of the drying chamber

Total energy input into drying chamber from both solar collector and direct solar irradiance is given by,

6 $$Ed_{{in}} = \left( {\tau \times \alpha \times I \times A_{c} } \right) + \left( {I \times A_{d} } \right)$$

Energy efficiency of the dryer is given as

7 $$\:{\eta\:}_{d}=\frac{{M}_{p}\times\:L}{{Ed}_{in}}$$

Where $\:{M}_{p}$ is moisture quantity removed from products in kg and $\:L$ is latent heat of fusion required to evaporate the moisture.

### Exergy analysis

The exergy analysis was performed according to the second law of thermodynamics, which assesses quality of the energy produced by the dryer system.The flow is assumed to be steady for this analysis and the changes due to kinetic, potential energy and pressure heads are neglected. The exergy balance equation is given by,

8 $$\:\sum\:{\dot{Ex}}_{in}-\sum\:{\dot{Ex}}_{out}=\sum\:{\dot{Ex}}_{loss}$$

For the analysis, the total exergy in flow and out flow of solar collector and the total exergy inflow and outflow of the dryer has to be considered. This is calculated using the simplified equation,

9 $$\:\dot{Ex}={{\dot{m}}_{a}C}_{p}\left[\left(T-{T}_{amb}\right)-{T}_{amb}ln\left(\frac{T}{{T}_{amb}}\right)\right]$$

#### Exergy analysis of the solar collector

Exergy inflow into collector is given by

10 $$\:{\dot{Ex}}_{Ci}=\left[1-\frac{{T}_{amb}}{{T}_{sun\:}}\right]{Ec}_{in}$$

Where $\:{T}_{sun\:}$is the apparent temperature of the sun (6000 K). The exergy outlet from the collector is

11 $$\:{\dot{Ex}}_{C0}={{\dot{m}}_{a}C}_{p}\left[\left({T}_{C0}-{T}_{Ci}\right)-{T}_{amb}ln\left(\frac{{T}_{Co}}{{T}_{Ci}}\right)\right]$$

Exergy efficiency is the ratio of exergy outflow to the exergy inflow

12 $$\:{\eta\:}_{ExC}=\frac{{\dot{Ex}}_{C0}}{{\dot{Ex}}_{Ci}}$$

#### Exergy analysis of the drying chamber

Exergy inflow into the drying chamber is calculated by

13 $$\:{\dot{Ex}}_{di}={{\dot{m}}_{a}C}_{p}\left[\left({T}_{di}-{T}_{amb}\right)-{T}_{amb}ln\left(\frac{{T}_{di}}{{T}_{amb}}\right)\right]+\left[1-\frac{{T}_{amb}}{{T}_{sun\:}}\right](I\times\:\alpha\:\times\:{A}_{d})$$

Exergy outflow from the drying chamber is given by

14 $$\:{\dot{Ex}}_{do}={{\dot{m}}_{a}C}_{p}\left[\left({T}_{do}-{T}_{amb}\right)-{T}_{amb}ln\left(\frac{{T}_{do}}{{T}_{amb}}\right)\right]$$

Exergy efficiency of drying chamber is

15 $$\:{\eta\:}_{ExD}=\frac{{\dot{Ex}}_{do}}{{\dot{Ex}}_{di}}$$

#### Exergy sustainability indicators

Thesustainability characteristics and potential of the dryer system is analysed through exergy sustainability indicators.

16 $$\:WER=\frac{{\dot{Ex}}_{dL}}{{\dot{Ex}}_{di}}$$

17 $$\:SI=\frac{1}{1-{\eta\:}_{ExD}}$$

18 $$\:IP=(1-{\eta\:}_{ExD}){\dot{Ex}}_{dL}$$

19 $$\:EIF=WER\frac{1}{{\eta\:}_{ExD}}$$

20 $$\:LOP=\frac{{\dot{Ex}}_{dL}}{{\dot{Ex}}_{do}}$$

21 $$\:EDF=\frac{1}{{\eta\:}_{ExD}}$$

### Environmental analysis

The dryer system is built by consuming energy during various stages of its life cycle, from material extraction, fabrication, operations and disposal. This cumulative energy consumed is subdivided as operational energy and embodied energy. For efficient renewable energy devices, embodied energy contributes greatly to the total life cycle energy consumption. Considering this embodied energy and operational energy, the environmental analysis of dryer system was performed in understanding energy payback period, CO_2_ mitigation potentialand the carbon credits earned.Table 4 lists the embodied energy of the individual components in the dryer system.

**Table 4:** Embodied energy of individual components of the solar dryer.

S no.	Component	Co-efficient of embodied energy (kWh/kg)	Quantity (kg)	Total embodied energy (kWh)
1.	Polycarbonate sheet	10.19	5	50.95
2.	Aluminium absorber plates	9.636	21	202.35
3.	Glass	7.27	5	36.35
4.	Mild steel components	8.9	14	124.6
5.	Drying trays	55.28	11.6	641.248
6.	Plywood	11.92	13	154.96
7.	Hinges	55.28	2	110.56
8.	Latches	55.28	0.5	27.64
9.	PVC pipes	0.34	3	1.02
10.	Paint	25.11	2	50.22
11.	Nuts and bolts	9.67	1	9.67
12.	Foam sheet	20.55	7	143.85
13.	Copper tubes	30	1.5	45
14.	Lauric acid	2.73	4	10.92
15.	Black pebbles	0.073	20	1.46
16.	Steel parts	8.89	8	71.12
	Total embodied energy			1681.918

#### Energy payback period

Energy payback period refers to the duration needed for dryer system to offset the energy consumed during its production through its output.

22 $$\:{E}_{PB}=\frac{Embodied\:energy\:\left(kWh\right)}{Annual\:Energy\:output\:(kWh/year)}$$

Where annual energy output of dryer is

$$\:{AE}_{out}\left(kWh\right)=Daily\:thermal\:output\times\:{D}_{n}$$

Where $\:{D}_{n}$denotes the total sunny days within a year in the location of dryer and is taken as300 considering the tropical climate of the location.

23 $$\:Daily\:thermal\:output\:\left(kWh\right)=\frac{Total\:moisture\:evaporated\:\left(kg\right)\times\:L}{3.6\times\:{10}^{6}}$$

#### CO2 emission from the solar dryer

When electricity is produced through conventional fuel like coal, the average emission of greenhouse gas is considered as 0.91 kg of CO_2_ per kWh. The CO_2_ emission by the dryer is calculated as

24 $$\:Annual\:{\text{C}\text{O}}_{2}\text{e}\text{m}\text{i}\text{s}\text{s}\text{i}\text{o}\text{n}=\frac{Embodied\:energy\times\:0.98}{Life\:time\:of\:dryer}$$

We consider the transmission losses (L_t_) to be 40% and losses due to old appliances (L_a_) as 20%, and a 25 years of life span for the dryer, in the equation,

25 $$\:Annual\:{\text{C}\text{O}}_{2}\text{e}\text{m}\text{i}\text{s}\text{s}\text{i}\text{o}\text{n}=\frac{Embodied\:energy}{Life\:time\:of\:dryer}\times\:\frac{1}{1-{L}_{a}}\times\:\frac{1}{1-{L}_{t}}\times\:0.98\:kg$$

The equation is simplified to,

26 $$\:Annual\:{\text{C}\text{O}}_{2}\text{e}\text{m}\text{i}\text{s}\text{s}\text{i}\text{o}\text{n}=\frac{Embodied\:energy}{Life\:time\:of\:dryer}\times\:2.042\:kg$$

#### CO2 mitigation potential

The CO_2_ mitigation potential by the solar dryer through its operation is expressed using the Eq. 

27 $$\:Annual\:{\text{C}\text{O}}_{2}\text{m}\text{i}\text{t}\text{i}\text{g}\text{a}\text{t}\text{i}\text{o}\text{n}=\left(Embodied\:energy\times\:Life\:time\:of\:dryer-{AE}_{out}\right)\times\:2.042\:kg$$

#### Carbon credits

Carbon credit denotes the emission prevention potential or absorption potential of the dryer system in terms of tons of CO_2_. This is given as

28 $$\:Carbon\:credits\:earned=Annual\:{\text{C}\text{O}}_{2}\text{m}\text{i}\text{t}\text{i}\text{g}\text{a}\text{t}\text{i}\text{o}\text{n}\:\text{i}\text{n}\:\text{t}\text{o}\text{n}\text{s}\times\:Cost\:of\:{\text{C}\text{O}}_{2}\text{m}\text{i}\text{t}\text{i}\text{g}\text{a}\text{t}\text{i}\text{o}\text{n}\:\text{p}\text{e}\text{r}\:\text{t}\text{o}\text{n}$$

### Uncertainty Estimation in the measured parameters

The accuracy of the instruments employed in the study leads to unavoidable uncertainties in the measured parameters, which indicate a possible variation from the obtained results.Table 5 gives the uncertainties of independent measuring parameters in the experiment. The uncertainty of a dependent parameter (M_R_) with independent variables x_1_, x_2_, x_3_ etc. which has their uncertainties as $\:{w}_{1}$, $\:{w}_{2},{w}_{3}$etc. is found using the equation and is tabulated in Table 6 ref^50^.

29 $$\:{M}_{R}={\left[{\left(\frac{{\partial\:}_{R}}{{\partial\:}_{x1}}{w}_{1}\right)}^{2}+{\left(\frac{{\partial\:}_{R}}{{\partial\:}_{x2}}{w}_{2}\right)}^{2}+{\left(\frac{{\partial\:}_{R}}{{\partial\:}_{x3}}{w}_{3}\right)}^{2}+\dots\:{\left(\frac{{\partial\:}_{R}}{{\partial\:}_{xn}}{w}_{n}\right)}^{2}\right]}^{\frac{1}{2}}$$

**Table 5:** Uncertainties in the independent measuring parameters.

S. no.	Parameter	Unit of measurement	Uncertainty
1.	Temperature measurement	ºC	± 0.21
2.	Solar radiation measurement	W/m^2^	± 2.1
3.	Mass of the product	kg	± 0.002
4.	Mass flow rate	m/s	± 0.3
5.	Data acquisition system	%	± 0.3

**Table 6:** Uncertainties of measured parameters in the experimental analysis.

S. no.	Measured parameter	Uncertainty (%)
1.	Useful energy gain of collector	± 1.46
2.	Collector energy efficiency	± 2.16
3.	Dryer energy efficiency	± 2.76
4.	Collector exergy efficiency	± 2.21
5.	Dryer exergy efficiency	± 2.28
6.	IP	± 3.18
7.	WER	± 2.25
8.	SI	± 2.25
9.	EIF	± 2.68
10.	LOP	± 2.56
11.	EDF	± 2.28

## Results and discussion

The experimental analysis was performed during months of July and August 2024 with moderate and fluctuating solar irradiance.The thermodynamic characteristics of the solar dryer were studied in the first section of the experiment followed by the emissions and CO_2_ mitigation capability.Further, the sustainability analysis of dryer was carried out from the exergy analysis results.

### Temperature profile of solar collector, dryer and solar irradiance

The experiment was carried out under forced convection without any thermal energy storage at first where the total drying time was 12 h which extended the experiment to the next day. Then SHES material was added at the base of the dryer and experiment was carried out with equal weight of product and this was completed within the same day, having a drying time of 9 h. To this experimental set up, a novel trapezoidal extended surface filled with Lauric acid was attached at the solar collector outlet, that acted at the LHES medium.The drying experiment was carried out and the desired moisture content was achieved within 6 h of operation. Considering the drying time of the Cucumis sativus slices under the open sun which was 18 h, the dryer system could achieve a drastic reduction in drying time. The temperature profile of the collector and drying chamber for each of these experimental cases are given in Fig. 6.

**Fig. 5:**
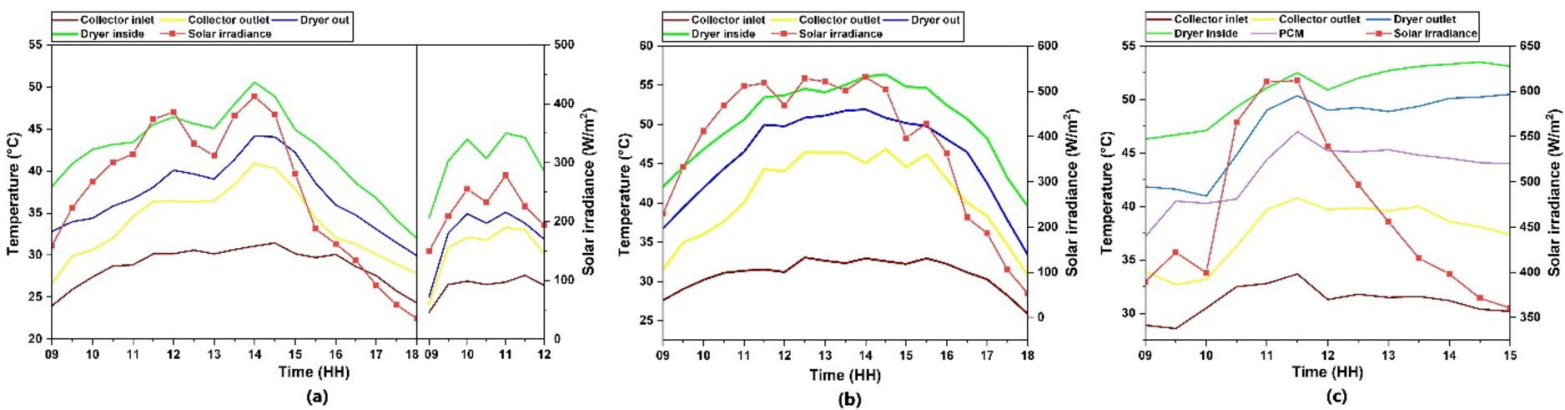
Temperature profile of the solar dryer (**a**) case 1 (**b**) case 2 (**b**) case 3.

During the experimental analysis, the solar irradiance varied between 150 and 612 W/m^2^ and was fluctuating in nature.An average irradiance of 326, 347 and 361 W/m^2^ was observed during the days of experiment for case 1, 2 and 3 respectively. In case 1 and case 2,outlet temperature from the solar collector aligned with the solar radiation intensity curve which is seen in Fig. 5a and Fig. 5(b)whereas in case 3, the LHES provision enabled to exhibit a consistent temperature output even under declining solar irradiance as seen in Fig. 5c. Drying chamber maintained an elevated temperature in all the cases, where a stable and most consistent curve was observed in case 2, which could be attributed towards the nature of solar irradiance along with the SHES provisioninside the dryer.Even towards final hours of dryingprocess in case 2, the drying chamber retains a higher temperature inside it indicating the release of stored excess energy from the SHES.The temperature inside drying chamber temperature to the ambient was highestin case 2 and the high solar irradiance resulted in highest recorded temperature at the collector output which was 47ºC.Case 3 saw the solar irradiance showing a steep decline as the experiment progresses, while the output from the collector as well as the dryer temperature remains elevated implying the effective storage and retrieval of thermal energy from SHES and LHES materials. In case 3, the temperature profiles show a susceptibility to the solar irradiance curve at the beginning of the experiment and the dryer temperature and collector outlet are relatively lower during this period when compared to case 1 and case 2. This occurs because some of the energy from the heated air out fromthe collector is transferred toPCM, resulting in a lowered output. The solar irradiance peaks towards the initial hours and then declines which enables retrieval of the stored energy that helps in maintaining the temperature profile, leading to the least drying time in the whole experiment.An effective heat transfer among PCM and hot air outlet of collector can be observed towards the final drying hours, leading to a temperature profile that is contrary to the solar irradiance curve. This indicates that the PCM inside the chamber has been charged with the incoming solar irradiance as well the hot air output from the collector during the initial hours of the experiment, which is then discharged into the air passing through it towards the final hours of drying.

### Energy analysis

**Fig. 6:**
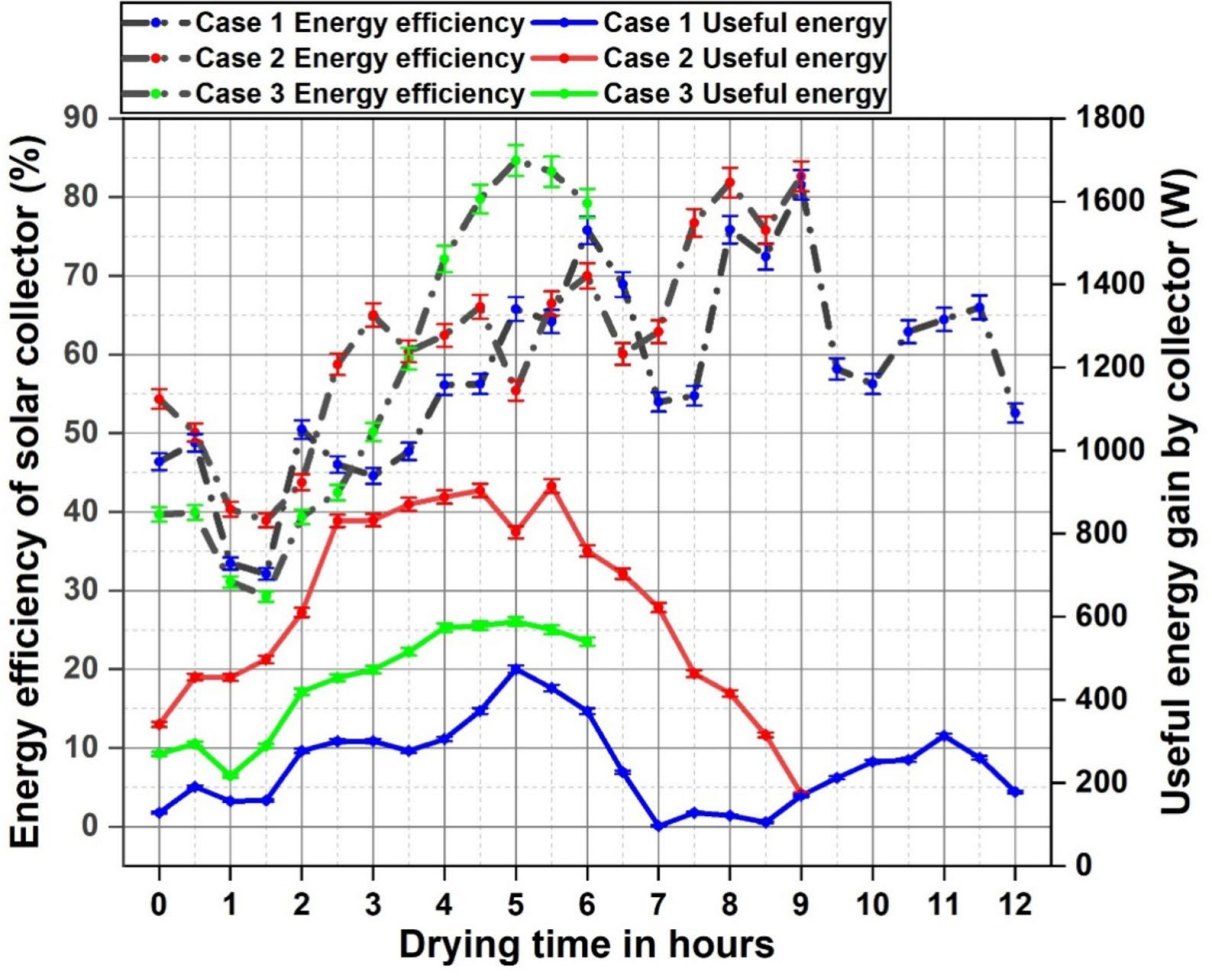
Useful heat gain and energy efficiency of the solar collector.

Solar collector is a crucialcomponent in the dryer system through which the heated air is properly channelled into the drying chamber and enables the removal of moisture filled air towardsdrying chamber outlet. The solar collector contributes significantly towards the elevated dryer temperature inside the dryer through a constant supply of hot air. The solar radiation incident on the surface of the absorber plate heats it up which is collected by the air flowing underneath it. This useful energy gain and solar collector energy efficiency is plotted in Fig. 6.Energy efficiency curve aligns with the useful heat gain profile and the plotted curves provides an insight into the effect of addition of energy storage materials in terms of thermal performance. Case 1 exhibited an average energy efficiency of 53.4%, whereas case 2 achieved an efficiency of 61%. case 3 reported an average thermal efficiency of 58%, which was lower than case 2 which can be attributed towards the difference in the number of hours operated. Energy gain by the collector shows the highest values in case 2 which is in accordance with the high solar irradiance during the experiment. With the highest energy gain value of 915 W during noon, an average of 624 W was reported for case 2 while this was 251 W for case 1 and 445 W for case 2.The final hours of the experiment post sunshine hours show a spike in the energy efficiency especially in case 1 and case 2 which is due to the fact that even though the useful energy gain is declining, the energy input from the solar irradiance is minimal to null at this point and the resultant temperature output is from the residual energy stored in the absorber plate. This leads to a slightly elevated temperature yet with minimal solar irradiance, which in turn reports a high thermal efficiency. Case 3 reports the least thermal performance in terms of energy efficiency during the initial hours of the experiment because of the lower temperature at the collector outlet. A part of the heat energy carried by the collector outlet air is transferred into the PCM leading to a decline in output temperature which is reflected in the energy efficiency curve. Yet this heat exchange is paid off in the later hours as a stable and elevated temperature is maintained at the collector output for hours irrespective of the declined solar irradiance. This is reflected as a spike in energy efficiency curve for case 3 withthe highest energy efficiency of the solar collector reported at 84.6%. In this experiment, case 1 and case 2 exhibited energy efficiency up to 81.5% and 82.6% respectively.Similar characteristic curve is observed in a study by Yematawu et al. where black pebbles were employed as an energy storage material in the solar collector, where the efficiency value went up to 62% towards the final hours^1^. With minimal to no solar irradiance during final hours of drying, the stored energy from SHES material contributed towards the elevated temperature at collector outlet, reporting high energy efficiency values during these hours. Anidentical comparative study with SHES and LHES materialsseparately was performed by Andharia et al. in a solar collector which also resulted in readings and efficiency curve similar to the obtained results in this study^51^. The energy efficiency values were similar and showed high values towards final hours of operation, which is also attributed towards the retrieval of stored energy during non-sunshine hours.

**Fig. 7:**
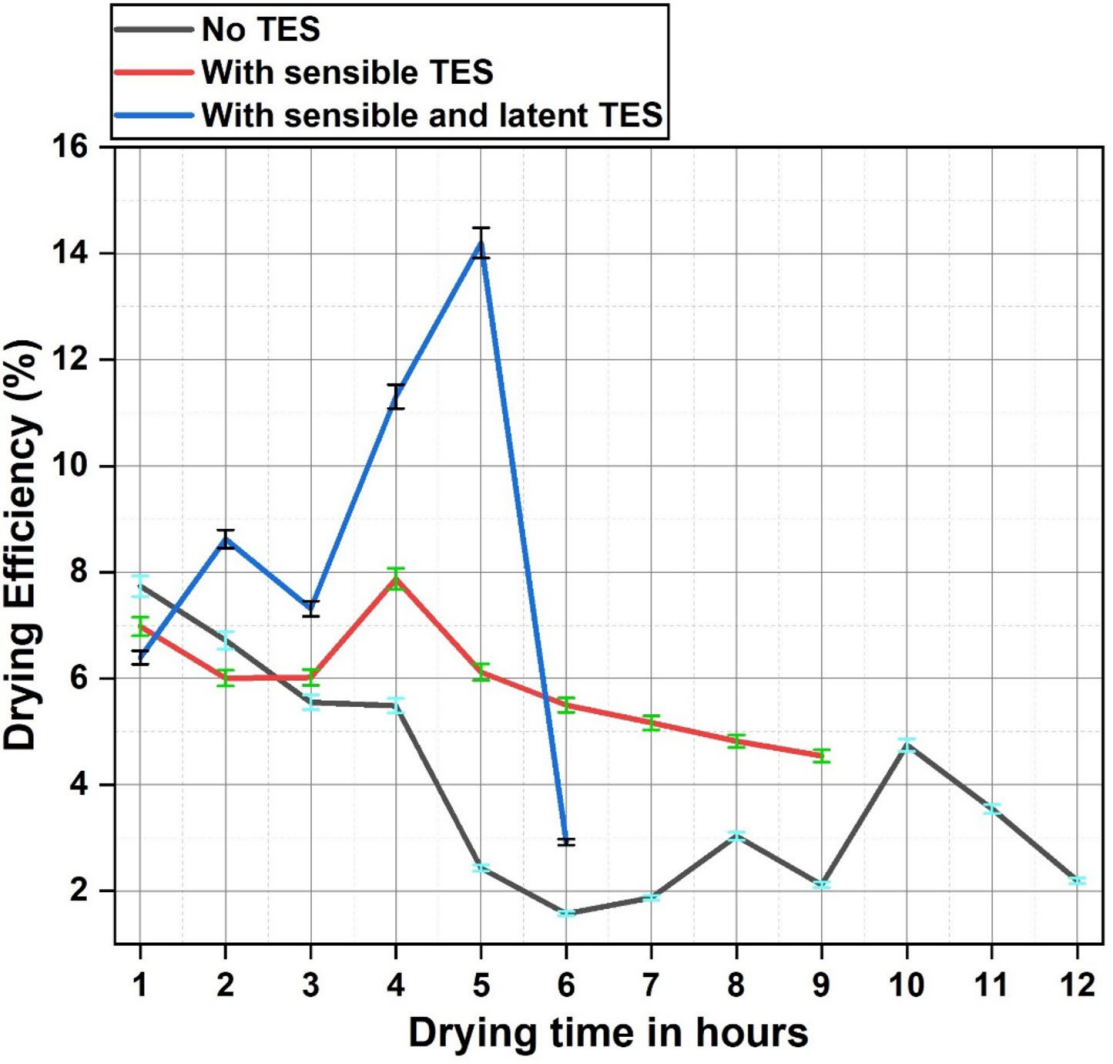
Energy efficiency of the solar dryer.

The solar dryer energy efficiency was evaluated taking into account the total energy received and supplied to the dryer, as well as the energy needed to evaporate moisture from products, which is plotted in Fig. 7.Case 1 exhibited the highest drying efficiency in the initial hour of the experiment, since the entire incoming irradiance was completely utilized for the drying purpose due to the absence of any thermal energy storage materials.The high drying efficiency during the initial hours is also attributed to the easier evaporation of unbound moisture present on the products surface. Even though the solar irradiance incident on the dryer is relatively high, this would also lead to increased losses and the entire input energy could necessarily not contribute for the drying purpose, which is observed in case 2. Similar trend was observed in case 3 where a high solar irradiance was reported during the initial hours yet the observed efficiency was relatively lower. Towardsfinal drying hours in case 2 and case 3, the drying of the products carried on due to the stored energy release from the energy storage materials resulting in a higher energy efficiency. Case 3 exhibited the highest energy efficiency of 14.2% and in case 2 the energy efficiency went up to 7.8%.The highest energy efficiency in case 1 was during the first hour of drying at 7.2%. Case 3 had an average energy efficiency of 8.5% which was the highest in the experiment. This was 5.9% for case 2 and 4% for case 1. Combined energy storage provision had definite advantages towardsthermal performancewhich can be seen in case 3 with a 53% increase in average efficiency from case 1.Gilago et al. performed a similar comparative analysis with only LHES in an indirect solar dryer for drying pineapple and the readings were similar with energy efficiency values within the vicinity of the results obtained in the present study. Dryer efficiency reported to be 10.84% with LHES whereas without energy storage this was 7.23%. The energy stored in the paraffin wax PCM contributes towards drying as the solar irradiance decreases, which exhibits the highest efficiency during drying. With most of the moisture removed from the products during this stage, the final hour of drying shows minimal efficiency since there isa only a small proportion of bounded moisture left in the product^16^. Another study by Mugi et al. by drying muskmelon slices in a solar dryer retains a maximum energy efficiency of 12.11% under forced convection, where the experiment was carried out under high solar irradiance compared to the conditions of present study^17^. This once again emphasizes the positive impact combined energy storage had in the thermal characteristics of the dryer in this experiment, even under moderate and fluctuating solar irradiance. Similar results in terms of energy efficiency were observed in other studies^52–54^. The release of stored energy in the SHES and LHES materials aided towards the moisture removal under declining solar irradiance which maintained the consistency of drying operation resulting in high efficiency observed in the study.

### Exergy analysis

**Fig. 8:**
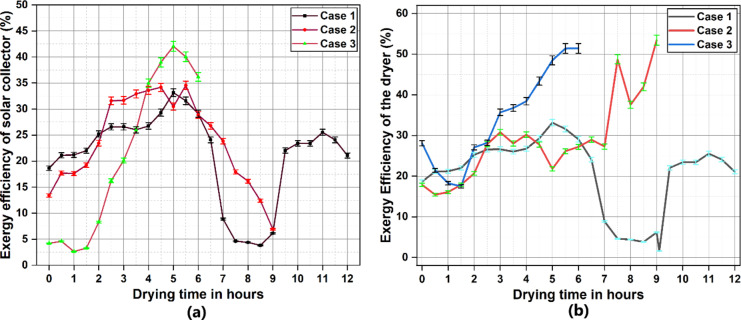
Variation in the exergy efficiency with respect to drying time (**a**) solar collector (**b**) solar dryer.

Exergy analysis inferred on quality of energy that is being collected and the reversibility of the process. The change in exergy efficiency of the solar collector in relation to drying time is illustrated in a plot in Fig. 8a.Exergy efficiency is dependent on the outlet air of the collector and incoming solar irradiance, as the mass flow rate remains constant. With no provision for thermal energy storage attached to the collector, the exergy profile of the collector in case 1 and case 2 was in alignment with the solar irradiance curve for the day.Exergy efficiency value peaked during solar noon for both case 1 and case 2 at 33.1%and 34.5% respectively. Case 1 had an average exergy efficiency of 21.1% and this was 23.8% for case 2. Being attached with an extended surface filled with PCM, the exergy characteristic curve of case 3 varied from the solar irradiance pattern for the day. The initial exergy efficiency was lower than case 1 and case 2 due to the lowered temperature output from the collector. Further as irradiance decreased, the outlet temperature was maintained at a higher value due to LHES, which is seen as a spike in the efficiency curve. This reported a 42% collector exergy efficiencywith an average efficiency of 21.35%.Exergy studies on similar flat plate solar collectors were reported such as the study by Kumar et al. where a solar dryer system attached to a flat plate solar collector which was tested using resin drying and observed an average exergy efficiency of 13%^55^. The current experimental results showed better values compared to the study which could be attributed to the addition of PCM at the collector outlet. The observed results from the exergy analysis was within the vicinity of readings recorded in previous studies with solar collectors integrated with LHES^56–58^.

**Fig. 9:**
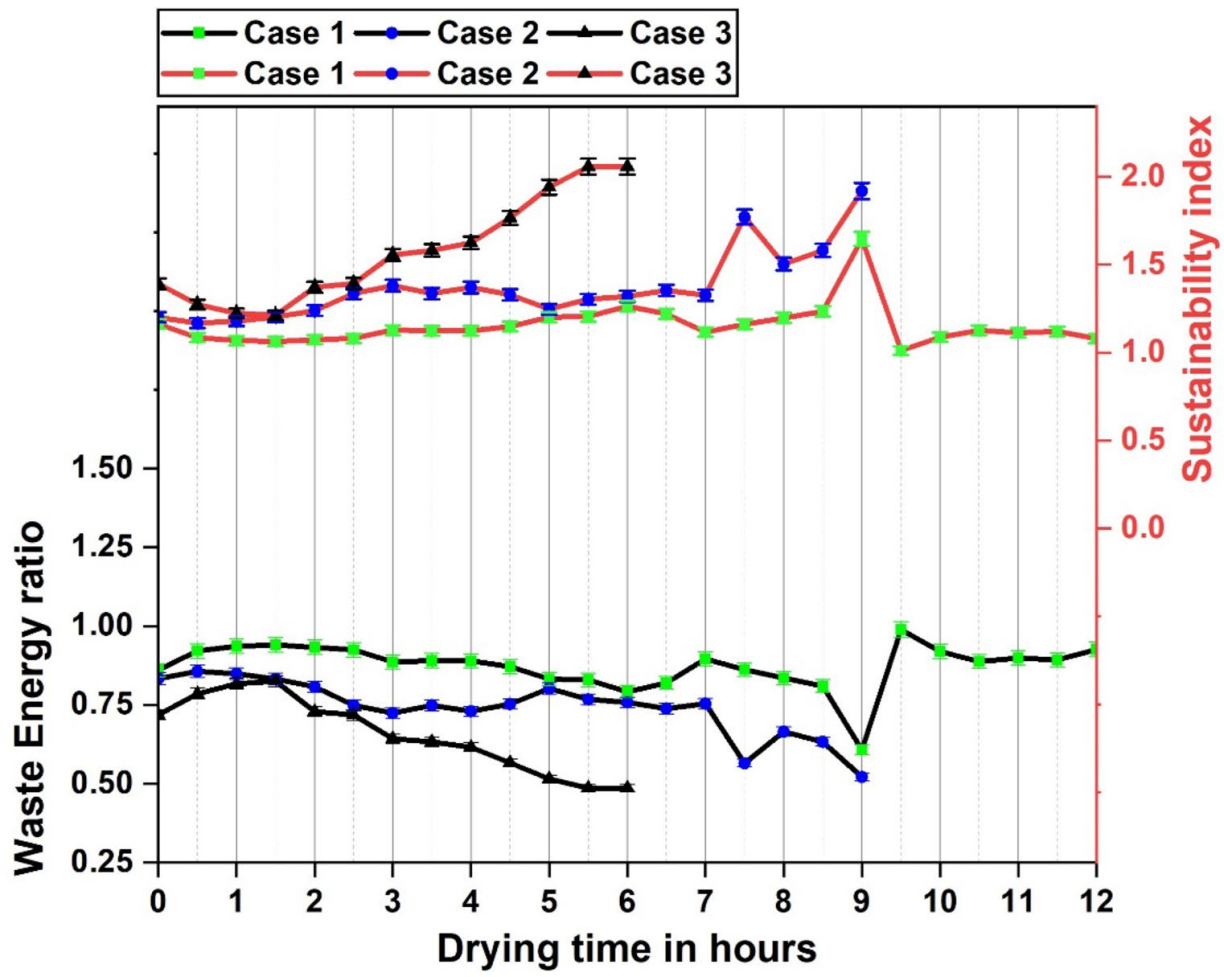
SI and WER of the solar dryer.

The exergy analysis of the drying chamber as plotted in Fig. 8(b)shows that case 1 follows a similar pattern in exergy efficiency where clear dependence on the solar intensity is visible. Compared to case 2 and case 3 the exergy efficiency at the initial hour of drying is highest in case 1 since no amount of energy is absorbed or transferred to an energy storage material. Exergy efficiency in case 1 goes as high as 33.1% for the drying chamber in solar noon and this gradually decreases towards the final hours, with an average of 20.3%. In case 2 and case 3, after a lower exergy efficiency during the initial hours a gradual increase can be observed where the credits can be given to the SHES and LHES. In case 2, the SHES materials absorbs a portion of the incoming solar irradiance to raise its own temperature, which is then released back into the drying chamber. This causes an elevation in temperature post sunshine hours inside the dryer leading to a higher exergy efficiency over final hours that went up to 53.44%. Case 3 has an even steeper spike in the exergy efficiency curve due to the combined effect of SHES and LHES. With the lowest exergy efficiency value during the initial hour of the experiment, the exergy efficiency sees a stable and constant climb in case 3 as observed in Fig. 9b indicating the release of stored energy directly into the drying chamber by SHES and through heated air by LHES materials. Case 3 exhibited highest average exergy efficiency at 34.3% and the peak exergy efficiency was recorded at 51.3%.These exergy values indicate that there is still a considerable amount of irreversibility that exists in the dryer system which could be due to the losses through reflections, joints, leakages, hinges, ducts etc.Similar results were observed in different research studies on solar dryers over the past few years^59,60^. Yacizi et al. performed a thermal analysis on a solar dryer reporting an exergy efficiency of 38.9%. The study had no provision for energy storage and the exergy was in close alignment with the solar irradiance and temperature curves^61^. An experimental analysis of a solar dryer equipped with PCM by Murugesan et al. exhibited an average exergy efficiency of 28%. Even though the observed values were lower than the present study, the usage of PCM was minimal and the placement of the PCM tubes were strategic to ensure maximum heat storage and retrieval, the study emphasizes the significance of method of integration of energy storage material in solar dryers^39^.

#### Sustainability analysis through exergy efficiency

SI, WER, IP, EIF, LOP and EDF known as sustainability indices, were calculated and plotted through the readings obtained from the exergy characteristics of the dryer, which aided in analysing the sustainability of the dryer system. The indices were learned through the exergy analysis of the dryer by taking into account the exergy losses observed in the dryer as well the exergy efficiency during the drying operation under different experimental cases. SI (sustainability index) is the measure of amount of exergy that is supplied into the dryer per unit loss of exergy and the reciprocal of the same is known as WER (waste energy ratio), that indicates the exergy loss per unit exergy supply into dryer. IP (improvement potential), as the name suggests, denotes the percentage of exergy per unit input that could potentially be utilized for improving the system. This infers on the amount of irreversibility that can be reduced for a better performance of the dryer.EIF (environmental impact factor) is linked to the exergy losses in the system such that lower the exergy losses, lower is the disturbance the system causes to the outside surroundings or atmosphere. The sustainability of a system is assessed through the productivity obtained from it and LOP(lack of productivity) is an indicator denoting the amount of energy that effectively interacted with the purpose and was utilized in the system. EDF(exergy destruction factor) denotes the amount of exergy that was destroyed in a system through irreversible heat transfer and thermal degradations^17,52,62^.

**Fig. 10:**
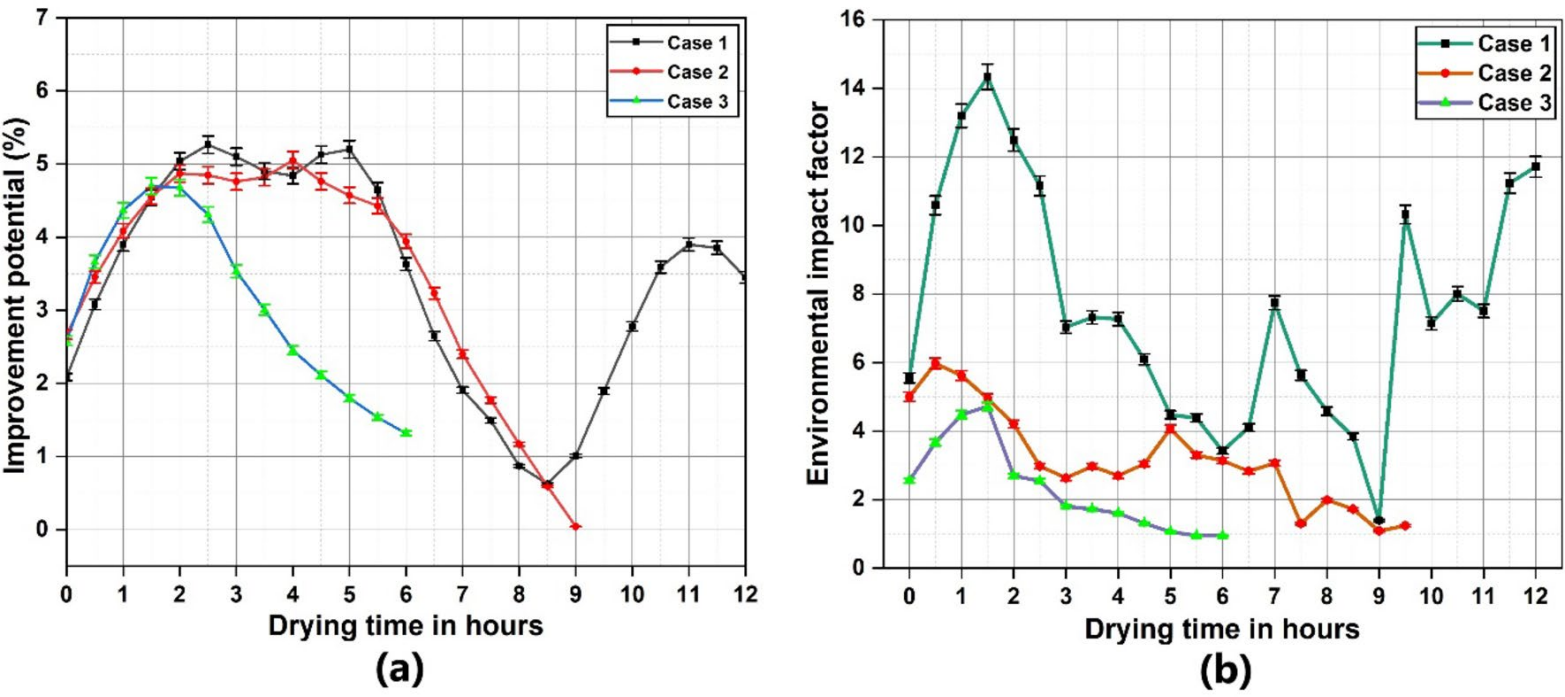
(**a**) Improvement Potential and (**b**) Environmental Impact Factor of solar dryer.

The SI and WER variation in the dryer with respect to drying time is plotted in Fig. 9.The SI values ranged between 1.01 and 1.23, 1.16–1.9 and 1.21–2.05 for case 1, 2 and 3, respectively whileWER values varied between 0.6 and 0.94, 0.52-0.85 and 0.48-0.82 for case 1, 2 and3, respectively. Highest WER was recorded in case 1 resulting in lower SI among all the cases.Provision of energy storage reflects significantly in the sustainability characteristics of the dryer, showing an improved SI with reduction in energy wastage. Case 3 exhibited the highest SI among all the experimental cases indicating clear sustainability benefits for combined energy storage. The variation in IP and EIF of the solar dryer for different cases is plotted in Fig. 10. The better utilization of the incoming energy through SHES and LHES reflects in the IP profile of the solar dryer for case 3, where the values range between 1.3–4.6%.Even with the provision of SHES in case 2, increased exergy losses resulted in an IP profile similar to that of case 1. The average values of IP were observed to be 3.4%, 3.2% and 3% forcase 1, 2 and 3, respectively.EIF observed during case 1 was considerably higher ranging between 1.2 and 14.33 with an average of 7.6, which is attributed towards higher exergy losses and absence of provision for energy storage. Case 2 had the highest EIF at 5.9 with an average of 3.1 whereas case 3 had the best results with an average of 2.3 and the highest EIF value was 4.7.The SI, IP and EIF values of the solar dryer obtained were within the vicinity of the results reported by Anuma et al. and Mugi et al. in similar studies on a greenhouse solar dryer and indirect solar dryer system, respectively^17,52^. The WER values of the solar dryer system were comparable to the readings reported by Ndukwu et al.^62^.

**Fig. 11:**
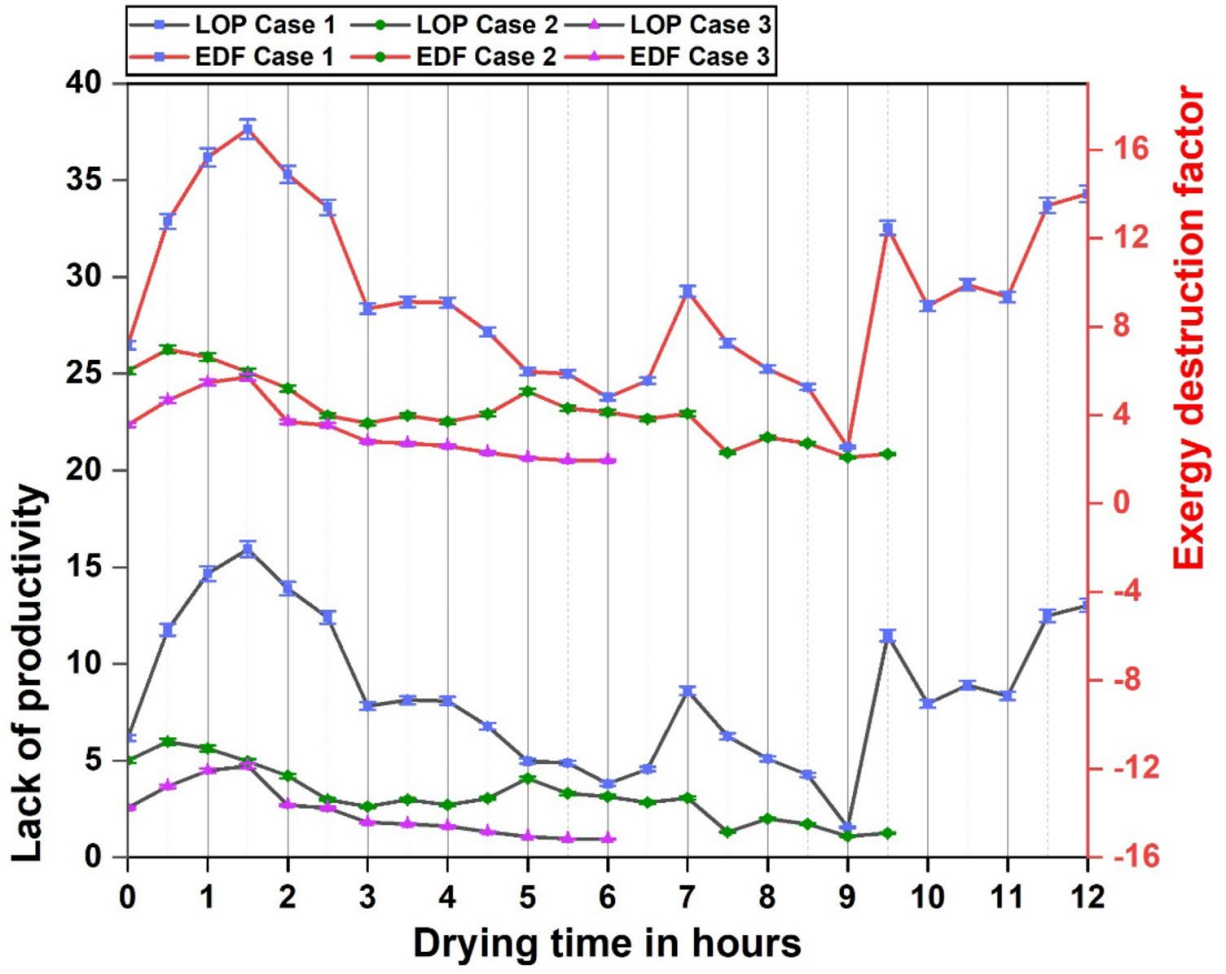
Lack of productivity and Exergy destruction factor of solar dryer.

The LOP and EDF profile of the solar dryer given in Fig. 11 depicts their corelation indicating that the energy lost in the dryer is in accordance with the exergy destruction through irreversible heat transfers including different losses. Case 1 exhibits higher losses and lack of production whereas case 2 and case 3 exhibits better energy utilization especially over the final hours. Energy storage and retrieval achieved better utilization of the incoming energy and could ensure better dryer performance.With the provision for combined energy storage, significant reduction was observed in WER, IP, EIF, LOP, EDF and proportional improvements in the SI. With both SHES and LHES, the dryer had an increment of SI by 39% was achieved in the dryer along with a reduction of WER, IP, EIF, LOP and EDF by 28%, 12%, 69%,72% and 66% respectively.An improved LOP and EDF values were achieved in this dryer compared to a previous study by Anuma et al. and Ndukwu et al.^52,62^.

### Environmental analysis

The solar dryer system was fabricated with low embodied commonly available materials which could easily be recycled. It required minimum amount of land space and there was minimal to none usage of water in the drying process, except for preprocessing of the products. Further, the energy payback period was calculated by taking into account the embodied energy of each individual components in dryer system.The emissions associated with the operation of dryer, its CO_2_ mitigation potential and the carbon credits earned during each operating case were determined. The least energy payback period for the dryer was calculated in case 3 which can be attributed towards the lower drying time and better thermal performance observed in this case. Case 3 reported a 1.82 years energy payback period while it was 2.49 years for case 2 and 3.09 years for case 1. The annual CO_2_ emissions of the solar dryer was 132.69, 136.48 and 137.37 kg/ year for case 1, 2 and 3 respectively. The highest CO_2_ mitigation potential of 83.97 tonnes per year was exhibited by case 3 while it was 83.93 and 81.85 tonnes per year for case 2 and case 1 respectively. The total carbon credits earned by the solar dryer during case 1 is 409$ and for case 2 and case 3 it was 419.65$ and 419.85$. The observed readings excelled previously published results of similar studies^63–66^, emphasizing the environmental benignity of the dryer system. Addition of both SHES and LHES into the dryer system could achieve an additional CO_2_ mitigation potential of 2.07 tonnes a year and a 41.7% reduction in energy payback period. It has to be noted that addition of energy storage materials contributes towards the total embodied energy of the system, yet this is compensated through enhanced performance and superior output, which results in lower energy payback period and higher CO_2_ mitigation potential.This underlines the environmental benefits of addition of energy storage materials into a renewable energy system.

## Conclusion

The thermal characteristics, sustainability and environmental analysis of a mixed mode solar dryer when integrated with both SHES and LHES were carried out in the study with Cucumis sativus as the product. The research performed under real time solar irradiance carried out a comparative analysis towards the effects of SHES, and SHES and LHES combined on the performance of the solar dryer. Significant performance enhancement was observed for the solar collector upon addition of the PCM chamber and in the solar dryer with the addition of PCM as well as black pebble stones. Combined thermal energy storage materials had highly advantageous impacts on the thermodynamic, environmental and sustainability characteristics of the solar dryer.The following conclusions were derived from the analysis.

- Addition of SHES could achieve a 25% reduction in drying time and addition of both SHES and LHES reduced the drying time by 50%.
- Integration of both energy storage materials achieved the highest energy efficiency of the dryer at 14.2% and resulted in a 53% increase in the average energy efficiency.
- Exergy analysis revealed superior exergy characteristics achieved through combined energy storage with highest exergy efficiency of 51.3%.
- Case 3 showed significant improvements in the exergy sustainability indices revealing better usage of incoming energy and lower impact on the environment. There was minimal energy wastage observed in this case with improved sustainability and higher productivity.
- Considering the life span of the dryer, it had a very low energy payback period of 1.82 years when operated with both energy storage materials.
- CO_2_ mitigation potential of 83.97 tonnes per year was reported by the solar dryer with a carbon credit of 419.85$.

This research should be carried forward by exploring integration of novel energy storage materials and analysing effects of placement of the energy storage materials and their quantity in overall performance of solar dryers. Ensuring a continuous operation of solar dryers irrespective of time and season through energy storage along with auxiliary heating sources increase their acceptability in the commercial sector. Policy making and raising awareness among the masses to acknowledge the potential of solar thermal devices is necessary in ensuring their desirability among rural populations.

## Electronic supplementary material

Below is the link to the electronic supplementary material.

Supplementary Material 1

## Data Availability

The datasets used and/or analysed during the current study available from the corresponding author on reasonable request.

## List of symbols

$\:{A}_{c}$: Area of solar air heater (m^2^)
$\:{A}_{d}$: Area of drying chamber (m^2^)
$\:{AE}_{out}$: Annual energy output of the dryer (kWh)
$\:{Ec}_{in}$: Energy input into the collector (W)
$\:{C}_{p}$: Specific heat capacity of air (kJ/kg K)
$\:{Ed}_{in}$: Solar energy incident on the dryer (W)
$\:{Edryer}_{total}$: Total energy input into the dryer (W)
$\:{\dot{Ex}}_{Ci}$: Exergy inlet into the collector (W)
$\:{\dot{Ex}}_{Co}$: Exergy outlet of the collector (W)
$\:{\dot{Ex}}_{di}$: Exergy inlet into the dryer (W)
$\:{\dot{Ex}}_{dL}$: Exergy loss in the solar dryer (W)
$\:{\dot{Ex}}_{do}$: Exergy outlet from the dryer (W)
$\:h$: Number of working hours of dryer in a day
$$\:I$$: Solar irradiance (W/m^2^)
$\:l$: Life span of dryer
$\:L$: Latent heat of fusion of water (J/kg)
$\:{\dot{m}}_{air}$: Air mass flow rate (kg/s)
$\:{M}_{p}$: Quantity of moisture evaporated (kg)
$\:{Q}_{c}$: Useful energy gain by the collector (W)
$$\:{T}_{amb}$$: Ambient temperature (°C)
$\:{T}_{di}$: Inlet temperature of dryer (°C)
$\:{T}_{do}$: Outlet temperature of dryer (°C)
$\:{T}_{ci}$: inlet temperature of solar collector (°C)
$\:{T}_{co}$: Outlet temperature of solar collector(°C)

## Greek symbols

$\:\tau\:$: Transmittance of the material
$\:\alpha\:$: Absorptance of the material
$\:\rho\:$: Density of air (kg/m^3^)
$\:{\eta\:}_{c}$: Energy efficiency of the solar air heater
$\:{\eta\:}_{d}$: Drying efficiency of the dryer
$\:{\eta\:}_{ExD}$: Dryer exergy efficiency
$\:{\eta\:}_{ExC}$: Collector exergy efficiency

## Abbreviations

EDF: Exergy destruction factor
EIF: Environmental impact factor
IP: Improvement potential
LHES: Latent heat energy storage
LOP: Lack of productivity
SHES: Sensible heat energy storage
SI: Sustainability index
WER: Waste energy ratio
