# Supplementary material for: Thermal and environmental analysis of Cucumis sativus drying in a mixed mode solar dryer with combined sensible and latent heat energy storage
Source: Sci Rep. 2025 Mar 5;15:7715. doi: 10.1038/s41598-025-91971-4 (PMC11882818; doi:10.1038/s41598-025-91971-4)
Supplement: Supplementary file 1 — Supplementary Material 1 [file 41598_2025_91971_MOESM1_ESM.docx]

The uncertainty of a dependent parameter (M_R_) with independent variables x_1_, x_2_, x_3_ etc which has their uncertainties as $w_{1}$, $w_{2},$ $w_{3}$etc. is found using the equation;

$M_{R}=\left[ \left( \frac{\partial_{R}}{\partial_{x1}}w_{1} \right)^{2}+\left( \frac{\partial_{R}}{\partial_{x2}}w_{2} \right)^{2}+\left( \frac{\partial_{R}}{\partial_{x3}}w_{3} \right)^{2}+\ldots\left( \frac{\partial_{R}}{\partial_{xn}}w_{n} \right)^{2} \right]^{\frac{1}{2}}$

The errors made during the experimental analysis is given as

$E_{R}={(W}_{R}/R)\times100$

$E_{En.useful}=Useful energy gain of solar collector$

$E_{R}=Overall uncertainty of the measuremet$

$E_{En.useful}=(\frac{W_{En,eff}}{En,eff})\times100$

$W_{En,useful}=Error of the observed value$

$W_{En,useful}=\left[ \left( Temperature \right)^{2}+\left( Temperature \right)^{2}+{(mass flow rate)}^{2} \right]^{1/2}$

$W_{En,useful}=\left[ {(.21)}^{2}+{(.21)}^{2}{+(0.3)}^{2} \right]^{1/2}=.422$

$E_{R}=\frac{.422}{290}\times100=$1.46%
